# Role of reactive oxygen species in regulating 27-hydroxycholesterol-induced apoptosis of hematopoietic progenitor cells and myeloid cell lines

**DOI:** 10.1038/s41419-022-05360-0

**Published:** 2022-10-31

**Authors:** Soo-Yeon Woo, Hansong Lee, Su Min Park, Hee-Seon Choi, Jayoung Kim, Munju Kwon, Jihyung Sohn, Ji Ho Nam, Hyung-Sik Kim, Parkyong Song, Ninib Baryawno, Yun-Hak Kim, Koanhoi Kim, Dongjun Lee

**Affiliations:** 1 Department of Convergence Medicine, School of Medicine, Pusan National University, Yangsan, 50612 Republic of Korea; 2 Department of Biomedical Informatics, School of Medicine, Pusan National University, Yangsan, 50612 Republic of Korea; 3 Department of Radiation Oncology, Pusan National University School of Medicine, Yangsan, Republic of Korea; 4 Department of Life Science in Dentistry, School of Dentistry, Pusan National University, Yangsan, 50612 Republic of Korea; 5 Childhood Cancer Research Unit, Department of Women’s and Children’s Health, Karolinska Institutet, Stockholm, 17177 Sweden; 6 Department of Anatomy, School of Medicine, Pusan National University, Yangsan, 50612 Republic of Korea; 7 Department of Pharmacology, School of Medicine, Pusan National University, Yangsan, 50612 Republic of Korea

**Keywords:** Stem-cell research, Haematopoietic stem cells, Leukaemia, Apoptosis

## Abstract

Oxysterols are oxygenated derivatives of cholesterol that contain an additional hydroxy, epoxide, or ketone group in the sterol nucleus and/or a hydroxyl group in the side chain of the cholesterol molecule. 27-Hydroxycholesterol (27HC) is a side-chain oxysterol that is oxygenated at the 27th carbon atom of cholesterol. The oxysterol (27HC) is produced via oxidation by sterol 27-hydroxylase (CYP27A1) and metabolized via oxysterol 7a-hydroxylase (CYP7B1) for bile acid synthesis in the liver. A previous study has demonstrated that treatment with the alternative Estrogen receptor alpha (ERα) ligand 27HC induces ERα-dependent hematopoietic stem cell (HSC) mobilization. In addition, *Cyp27a1*-deficient mice demonstrate significantly reduced 27HC levels and HSC mobilization. Here, we report that exogenous 27HC treatment leads to a substantial reduction in the hematopoietic stem and progenitor cell (HSPC) population owing to significantly increased reactive oxygen species (ROS) levels and apoptosis in the bone marrow (BM). However, 27HC does not influence the population of mature hematopoietic cells in the BM. Furthermore, exogenous 27HC treatment suppresses cell growth and promotes ROS production and apoptosis in leukemic cells. Moreover, acute myeloid leukemia (AML) patients with high *CYP7B1* expression (expected to have inhibition of 27HC) had significantly shorter survival than those with low *CYP7B1* expression (expected to have an elevation of 27HC). Single-cell RNA-sequencing (scRNA seq) analysis revealed that the expression of *CYP7B1* was significantly increased in AML patients. Thus, our study suggests that 27HC may serve as a potent agent for regulating pools of HSPCs and may have an application as a novel therapeutic target for hematological malignancies. Collectively, pharmacological inhibition of CYP7B1 (expected to have an elevation of 27HC) would potentially have fewer long-term hematological side effects, particularly when used in combination with chemotherapy or radiation for the treatment of leukemia patients.

## Introduction

Cholesterol is a lipid that supports organismal health and is biosynthesized through metabolism [1]. It is an essential component of cell membrane [2] and regulates plasma membrane fluidity [3]. Owing to its hydrophilic and hydrophobic regions, cholesterol plays an important role in regulating membrane fluidity [4]. Cholesterol is also essential for the biosynthesis of steroid hormones, bile acid [5], and vitamin D [6, 7]. In addition, the body relies on a complex homeostatic network to modulate the balance of cholesterol levels [8]. It has been reported that an increase in circulating lipoprotein levels in the blood leads to accumulation in the subendothelial space and causes atherosclerosis [2]. Nearly 50 % of all heart attacks and strokes occur in patients with normal cholesterol levels [9]. Furthermore, oxidative stress and micro-inflammation are more complex and intimately linked with cardiovascular disease [10].

Oxysterols are molecules comprising 27 carbon atoms and are produced by the oxidation of cholesterol [11]. They are biologically active molecules that are produced by a variety of cells. Oxysterols play significant roles in both the immune and hematopoietic systems. They have been documented to exert cytotoxic, oxidative, and inflammatory effects [12], and/or immunosuppressive [13] effects on several cells. Several studies have reported the accumulation of oxysterols in various tissues during pathological processes, such as atherosclerosis [14–16], inflammation [17], trauma [18], oxidative stress [19], coronary heart disease [20], Alzheimer’s disease [21, 22], cataract formation [23], signal transduction, and immune function [24]. Furthermore, oxysterols are also generated during osteogenic differentiation of the BM [25].

27-Hydroxycholesterol (27HC) is the most abundant oxysterol in the blood of healthy individuals [26] and is generated from cholesterol by the sterol hydroxylase CYP27A1, which is abundant in the liver [27]. 27HC acts as a competitive ligand for the liver X receptor (LXR), which results in the activation of LXR [28, 29]. The level of 27HC in plasma correlates with total cholesterol levels [30]. Several studies have shown that 27HC has various functions. Elevations in 27HC via *Cyp7b1* deletion promote atherosclerosis in *Apoe*^−/−^ mice [31]. 27HC promotes atherosclerosis via proinflammatory processes mediated by estrogen receptor alpha (ERα). In monocytes/macrophages, 27HC upregulates pro-inflammatory genes and increases adhesion via estrogen receptor alpha (ERα) [32]. In the vasculature, it acts as an antagonist of ERα action and promotes cancer cell growth, metastasis, and atherosclerosis progression via inflammatory processes mediated by estrogen receptor alpha (ERα) [26, 31]. In addition, it is associated with a variety of cancers, including prostate cancer [33], and glioblastoma [34]. 27HC increases metastasis of other solid tumor types [35]. 27HC pretreatment increased the number of metastatic nodules in the lungs when syngeneic breast cancer cells (Met1 and E0771), colorectal cancer cells (MC38), lung cancer cells (Lewis Lung), melanoma (B16-F0), or pancreatic cells (KPC915) were injected [35]. 27HC has also been reported to permeate the blood-brain barrier and its augmented levels are associated with the impairment of neuronal morphology [36].

A recent study has demonstrated that 27HC induces hematopoietic stem cell (HSC) mobilization from BM to spleen, depending on the presence of estrogen receptors (ERα) and extramedullary hematopoiesis during pregnancy [37]. 27HC is an abundant oxysterol in steady-state blood that plays a critical role in maintaining the cardiovascular system and modulates its function [3, 38]. It is an endogenous selective estrogen receptor modulator (SERM) [38]. LDL increases the proliferation of hematopoietic stem and progenitor cells and myeloid cell differentiation [39]. However, the correlation between 27HC levels and hematopoiesis remains unknown. In this study, we have shown that exogenous 27HC treatment results in reduced hematopoietic stem and progenitor cell (HSPC) population, but does not influence the population of mature hematopoietic cells. In addition, 27HC treatment increases cell death and reactive oxygen species (ROS) levels in HSPCs. Furthermore, exogenous 27HC treatment also suppresses cell growth and promotes apoptosis in leukemic cells. Collectively, our findings suggest that 27HC critically affects the HSPC pool and may serve as a novel therapeutic target for hematological malignancies.

## Materials and methods

### Cell line and reagents

Human HL60, K562, and KG1α cells were purchased from the American Type Culture Collection (ATCC, VA, USA). These cells were maintained in RPMI medium supplemented with 10% fetal bovine serum (FBS) and penicillin/streptomycin. Primary BM cells were harvested from 3–5 female mice, isolated by crushing the femur and tibia bones in 2% FBS/PBS, RBC lysed, and cells passed through 40 µm or 70 µm nylon cell strainers (BD). Primary BM cells were cultured for 24 h in RPMI supplemented with 10% FBS, penicillin/streptomycin, stem cell factor (SCF; 5 ng/mL), IL3 (10 ng/mL), and IL6 (10 ng/mL) [40]. Cholesterol and 27OHChol were purchased from Sigma-Aldrich (Merck KGaA, MA, USA) and Santa Cruz Biotechnology, Inc (TX, USA), respectively. 10^7^ primary BM cells are treated with 13 µM Cholesterol, 0.62 µM, or 6.2 µM 27OHChol for 24 h or 48 h, respectively. 3 × 10^5^ HL60, K562, and KG1α myeloid leukemic cells are treated with 13 µM Cholesterol or 6.2 µM 27OHChol for 48 h, respectively.

### Animals

All animal experiments were conducted with the approval of the Pusan National University School of Medicine. C57BL/6J (CD45.2) and B6. SJL-Ptprca Pep3b/BoyJ (B6.SJL, CD45.1) was purchased from the Jackson Laboratory (ME, USA). For BM transplantation, lethally irradiated recipient mice were intravenously (i.v.) transplanted with competitor BM cells (3 × 10^6^) from CD45.2 mice and control vehicle, pre-treated cholesterol, and pre-treated 27HC test BM cells from CD45.1 mice (3 × 10^6^).

### Flow cytometric analysis

Flow cytometry was performed as described previously with antibodies listed in Supplemental Material [40]. Briefly, 10^7^ BM cells were collected from femurs and tibias of mice by flushing with fluorescence-activated cell sorting (FACS) buffer consisting of phosphate-buffered saline (PBS), 2% FBS, and penicillin/streptomycin. 7AAD was included as a viability dye for identifying the dead cells. Flow cytometry data were acquired on a BD FACSCanto 2 flow cytometer and analyzed using FlowJo software (Tree Star, Inc., OR, USA). Flow sorting was performed using a BD Aria 2 flow cytometer. Intracellular phospho-protein staining was performed as described previously [40]. Briefly, cells were incubated with primary anti-pIRE1α and peIF2α antibodies (Cell Signaling Technologies) in FACS buffer, which consisted of phosphate-buffered saline (PBS) containing 2% FBS for 30 min at 4 °C. The cells were washed and incubated with Alexa Fluor 488-conjugated secondary antibody (Invitrogen) for 30 min at 4 °C.

### Measurement of ROS and cell death

Intracellular ROS and apoptosis assays were performed as described previously [41]. Briefly, ROS levels were assessed by staining with 2′,7′-dichlorofluorescin diacetate (DCFDA; Invitrogen, Thermo Fisher Scientific, MA, USA) at a concentration of 20 mM for 30 min at 37 °C. Apoptosis was determined by staining with an AnnexinV staining kit (BD Pharmingen, NJ, USA). To analyze the ROS by flow cytometry, cells were first stained with antibodies for surface markers and then incubated DCFDA (20 mM) for 30 min at 37 °C. For analysis of apoptosis, cells were first stained with antibodies for surface markers and then incubated FITC-labeled annexin V (5 µl) and 7-amino-actinomycin D (7AAD) (BD Pharmingen) were added to cells resuspended in annexin V binding buffer for 20 min at RT. FACS analysis was performed with a FACSCanto 2 (BD).

### Reverse transcription-quantitative PCR (RT-qPCR)

RNA/cDNA isolation/syntheses were performed as described previously [40]. Briefly, total RNA was isolated from cells using QIAGEN RNeasy-Plus mini-columns according to the manufacturer’s protocol (Qiagen, Inc., Hilden, Germany) and was used to synthesize cDNA using a cDNA synthesis kit (SmartGene). cDNA was amplified using SYBR Green Q-PCR Master Mix (SmartGene) and ABI QuantStudio3 (Applied Biosystems). RT-qPCR was performed using the primer pairs with the following sequences: *Erα*_F: 5′-TTG TGT GCC TCA AAT CCA TC-3′, *Erα*_R: 5′-GAG ATG CTC CAT GCC TTT GT-3′; *Bax*_F: 5′-AGC AAA CTG GTG CTC AAG GC-3′, *Bax*_R: 5′-CCA CAA AGA TGG TCA CTG TC-3′; *Gapdh*_F: 5′-GCA CAG TCA AGG CCG AGA AT, *Gapdh*_R: 5′-GCC TTC TCC ATG GTG GTG AA. *GAPDH*_F: 5′-TGT TGC CAT CAA TGA CCC CTT-3′, *GAPDH*_R: 5′-CTC CAC GAC GTA CTC AGC G-3′.

### Assessment of ER stress

To analyze the ER stress response by RT-qPCR and intracellular phospho-protein staining. RT-qPCR was performed using the primer pairs with the following sequences: *Chop*_F: 5′-CAT GTT GAA GAT GAG CGG GTG-3′, *Chop*_R: 5’-TGG AAC ACT CTC TCC TCA GGT-3′; *Ire1*_F: 5′-CTT GAG GAA TTA CTG GCT TCT CA-3′, *Ire1*_R: 5′-TCC AGC ATC TTG GTG GATG-3′; and *Xbp1s*_F: 5′-GAG TCC GCA GCA GGT G-3′, *Xbp1s*_R: 5′-GTG TCA GAG TCC ATG GGA-3′. Intracellular phospho-protein staining was performed with anti-peIF2α antibody (Cell Signaling Technology, Inc., MA, USA).

### In silico analysis of *CYP7B1* in AML patients

Kaplan–Meier survival curves for *CYP7B1* were obtained using the tools at https://easysurv.net/#/app/home based on the GSE12417 data set (log-rank test, *p* = 0.00982) [42]. Individuals with AML patients were subdivided based on median *CYP7B1* expression levels (high expression (*n* = 56) and low expression (*n* = 55)) in tumor cells.

### Public scRNA-seq dataset and processing

The scRNA seq dataset of bone marrow (BM) cells was obtained from Gene Expression Omnibus (GEO) database with accession number GSE116256 [43]. The samples included 16 AML patients and five healthy donors. In the quality control step, The data was filtered to have at least 1000 transcripts and 500 unique genes also less than a 20% mitochondrial RNA percentage ratio. Data were integrated and analyzed using the R package ‘Seurat’ (version 4.0.4) [44]. Raw read counts were normalized and scaled by NormalizeData and ScaleData functions. Highly variable genes and integration features were identified using the default setting of the FindVariableFeatures function and SelectIntegrationFeatures function. For integrating, we applied reciprocal PCA called the ‘RPCA’ method since cells are comprised of heterogeneous status which is malignant and normal. Cells that are not clearly defined to be normal or malignant were excluded for further analysis. Then, principal component analysis (PCA) and t-distributed stochastic neighbor embedding (tSNE) were implemented for dimension reduction and visualization.

### Differential gene correlation analysis

To dissect differentially correlated genes between malignant and normal cells, we aggregated cell-level gene expression into sample-level pseudo-bulk gene expression. For differential gene correlation analysis (DGCA), we utilized the averaged pseudo-bulk data and conducted the Pearson correlation coefficient of ddcorAll() function in each cell type [45]. The correlated gene pairs with a p-value less than 0.05 and satisfying the following requirement were considered to be in a significant relationship: (1) case where no significant correlation in one condition, but either positive or negative relationship in the other condition and (2) case where negative correlation in one condition whereas positive correlation in the other condition.

### Gene ontology enrichment analysis of differentially correlated genes

We conducted gene ontology enrichment analysis for biological process (BP) through the database for annotation, visualization, and integrated discovery, DAIVD (version 6.8) [46]. Biological processes were selected based on *p* values smaller than 0.05 and the top 10 processes were shown.

### Statistics

The sample sizes required for the experiments were estimated based on the preliminary results. No blinding or randomization was performed in any of the experiments. The statistical significance of differences between population means was assessed using a two-tailed unpaired Student’s *t* test. Statistically significant differences (*p ≤ 0.05, **p ≤ 0.01, and ***p ≤ 0.001) for pairwise comparisons between the indicated data points are shown. For the scRNA-seq data, The statistical significance between the two groups was decided by a two-sided Wilcoxon rank-sum test with a threshold p-value of 0.05.

## Results

### 27HC affects the population of immature stem and progenitor cells but not the mature multi-lineage cells of the hematopoietic system

**Fig. 1:**
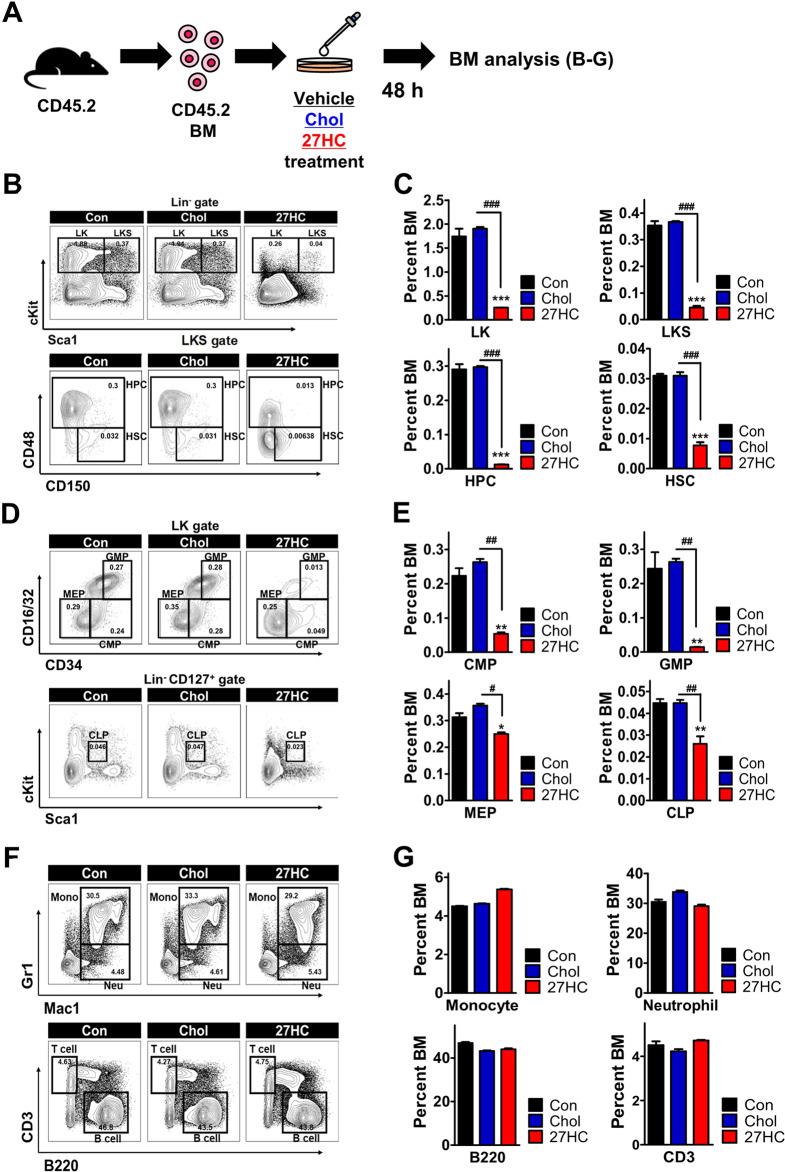
The exogenous addition of 27HC depletes hematopoietic stem and progenitor cells (HSPCs). BM cells are treated with 13 µM Cholesterol or 6.2 µM 27OHChol for 48 h, respectively. **A** Study overview. **B** FACS plot showing the frequency of LK, LKS, HPC, and HSC populations after 27HC treatment. **C** The frequencies of Lin^−^Sca1^−^cKit^+^ cells (LK), Lin^−^Sca1^+^cKit^+^ cells (LKS), HPCs (Lin^−^Sca1^+^cKit^+^ CD48^+^), and HSCs (Lin^−^Sca1^+^cKit^+^CD150^+^CD48^-^, SLAM cells) were decreased in the BM cells after exogenous 27HC treatment. **D** FACS plot showing the frequency of CMP, GMP, MEP, and CLP populations after 27HC treatment. **E** The frequencies of common myeloid progenitor (CMP) cells (Lin^−^Sca1^−^cKit^+^CD34^+^CD16/32^-^), granulocyte-macrophage progenitor (GMP) cells (Lin^−^Sca1^−^cKit^+^CD34^+^CD16/32^+^), megakaryocyte-erythroid progenitor (MEP) cells (Lin^−^Sca1^−^cKit^+^CD34^−^CD16/32^−^), and common lymphoid progenitor (CLP) cells (Lin^−^ Sca1^low^cKit^low^ CD127^+^) were decreased in the BM cells after exogenous 27HC treatment. **F** FACS plot showing the frequency of monocytes, neutrophils, B cells, and T cells after 27HC treatment. **G** The frequencies of monocytes (CD11b^+^ cells), neutrophils (CD11b^+^Gr1^+^ cells), B cells (B220^+^ cells) and T cells (CD3^+^ cells) were observed after 27HC treatment. Data are presented as mean ± SEM. (*p ≤ 0.05, **p ≤ 0.01, and ***p ≤ 0.001 vs. control; ^#^*p* ≤ 0.05, ^##^*p* ≤ 0.01, and ^###^*p* ≤ 0.001 vs. Chol). (*n* = 2 independent experiments and 3 total measurements per treatment).

The role of 27HC in hematopoiesis remains unknown. To delineate the function of 27HC, we first examined the level of *Erα* expression after 27HC treatment in HSPCs (Fig. S1A). We hypothesized that 27HC functions as an endogenous selective estrogen receptor modulator (SERM) in HSPCs, and *Erα* expression is increased in 27HC-treated Lin^−^Sca1^+^cKit^+^ cells (LKS) and HPCs (Lin^−^Sca1^+^cKit^+^ CD48^+^) (Fig. S1A). Our results revealed that normal HSPC indeed exhibits an increased expression of ERα in response to 27HC treatment. We have previously explored the molecular pathways affected by 27HC treatment in THP1 cells [47]. To assess whether 27HC affects HSPC, we exposed HSPCs in BM cells to 6.2 µM 27HC for 24 h (Fig. S2A) or 48 h (Fig. 1A–G), respectively. Interestingly, our analyses revealed that the exogenous 27HC treatment of BM cells led to a decrease in Lin^−^Sca1^-^cKit^+^ cells (LK) (Figs. 1B, C, and S2A), Lin^−^Sca1^+^cKit^+^ cells (LKS) (Figs. 1B, C, and S2A), HPCs (Lin^−^Sca1^+^cKit^+^ CD48^+^) (Figs. 1B, C, and S2A), HSCs (Lin^−^Sca1^+^cKit^+^CD150^+^CD48^−^, SLAM cells) (Figs. 1B, C, and S2A), common myeloid progenitor (CMP) cells (Lin^−^Sca1^-^cKit^+^CD34^+^CD16/32^−^) (Fig. 1D and E), granulocyte-macrophage progenitor (GMP) cells (Lin^−^Sca1^−^cKit^+^CD34^+^CD16/32^+^) (Fig. 1D, E), megakaryocyte-erythroid progenitor (MEP) cells (Lin^−^Sca1^−^cKit^+^CD34^−^CD16/32^−^) (Fig. 1D, E), and common lymphoid progenitor (CLP) cells (Lin^−^ Sca1^low^cKit^low^ CD127^+^) (Fig. 1D, E). In addition, no differences in the numbers of mature lineage cells such as monocytes (CD11b^+^ cells) (Fig. 1F, G), neutrophils (CD11b^+^Gr1^+^ cells) (Fig. 1F, G), B cells (B220^+^ cells) (Fig. 1F, G), and T cells (CD3^+^ cells) (Fig. 1F, G) were observed after the 6.2 µM 27HC treatment for 48 h. Moreover, no differences in the numbers of HSPC and mature lineage cells were observed after the 0.62 µM 27HC treatment for 24 h (Fig. S2B). Interestingly, we did observe decreased expression of cKit and CD48 in HSPC (Fig. S2C). cKit is the surface receptor of SCF and is essential for HSC self-renewal, growth, and survival [48]. Down-regulation of the cKit surface antigen could contribute to the impairment of self-renewal of HSPC in BM [49–51]. These results suggest that exogenous 27HC treatment results in impaired immature HSPC population, including cKit^+^ HSPCs, without influencing the mature hematopoietic lineage cell population in a time- and dose-dependent manner.

### Sequential augmentation of ROS, endoplasmic reticulum (ER) stress, and apoptosis is responsible for the depletion of HSPCs post 27HC treatment

**Fig. 2:**
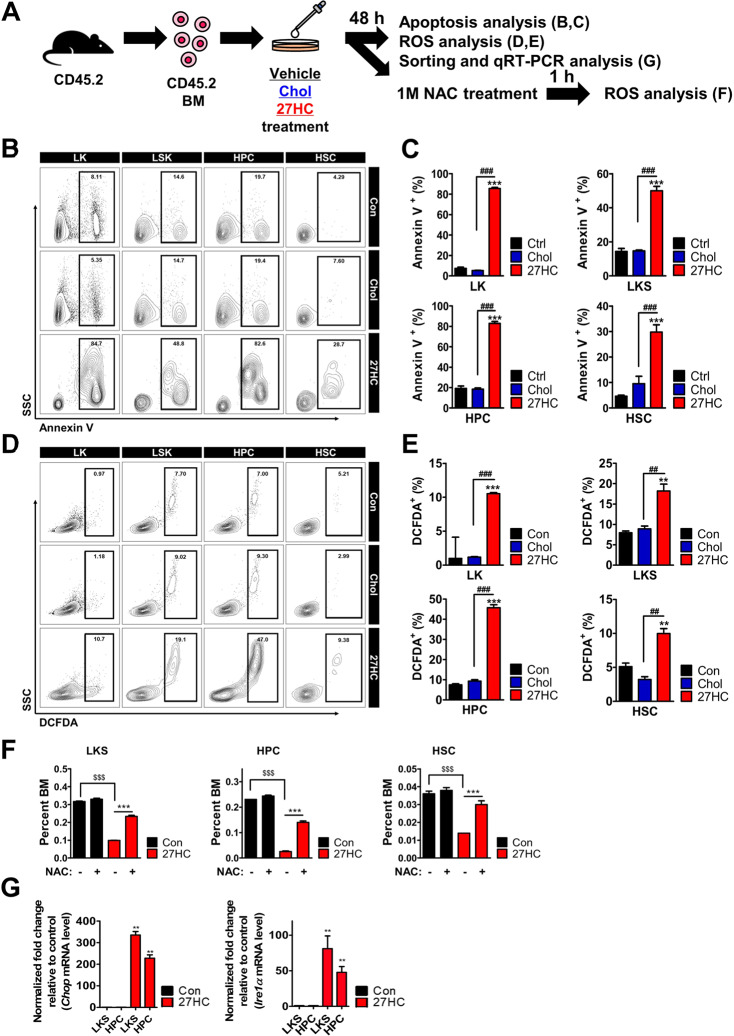
Exogenous addition of 27HC increases apoptosis and reactive oxygen species (ROS) in HSPCs. BM cells are treated with 13 µM Cholesterol or 6.2 µM 27OHChol for 48 h, respectively. **A** Study overview. **B** FACS plot showing the frequency of apoptosis of LK, LKS, HPC, and HSC populations after 27HC treatment. **C** The exogenous 27HC-treated LK, LKS, HPCs, and HSCs showed greater proportions of apoptotic cells than did control cells. **D** FACS plot showing ROS production in LKS, HPC, and HSC populations after 27HC treatment. **E** The exogenous 27HC-treated LK, LKS, HPCs, and HSCs showed greater ROS production than control cells. **F** The frequencies of Lin^−^Sca1^+^cKit^+^ cells (LKS), HPCs (Lin^−^Sca1^+^cKit^+^ CD48^+^), and HSCs (Lin^−^Sca1^+^cKit^+^CD150^+^CD48^−^, SLAM cells) were rescued in the BM cells after 1 mM NAC treatment. **G** BM cells are treated with 13 µM Cholesterol or 6.2 µM 27OHChol for 48 h, respectively. The relative levels of *Chop and Ire1a* mRNA were assessed from sorted LKS and HPC cells. *Chop and Ire1a* expression were increased in 27HC-treated LKS and HPCs compared to control. Data are presented as mean ± SEM. (**p ≤ 0.01 and ***p ≤ 0.001 vs. control; ^##^*p* ≤ 0.01 and ^###^*p* ≤ 0.001 vs. Chol; ^$$^*p* ≤ 0.01 and ^$$$^*p* ≤ 0.001 vs. with NAC treatment). (*n* = 2 independent experiments and 3 total measurements per treatment).

To determine the cause of decreased HPSCs post 27HC exposure, we analyzed whether altered apoptosis could contribute to the depletion of HSPCs after 27HC treatment in the BM cells (Fig. 2A–C). Our results revealed that there is an increase in the number of apoptotic (Annexin V^+^) cells in the LK, LKS, HPC, and HSC compartments after 27HC treatment (Fig. 2B, C). Subsequently, we further checked *Bcl-2*-associated X protein (*Bax*) expression in the LKS and HPC compartments (Fig. S1B). Our results showed that the expression of *Bax* was indeed augmented in the LKS and HPC compartments after the treatment of BM cells with 27HC. These data indicate that exogenous 27HC affects HSPC pools in vitro through the regulation of apoptosis.

Accordingly, we further assessed the ROS activity after 27HC treatment by measuring 2′,7′-dichlorofluorescein diacetate (DCFDA) from HPCs and HSCs (Fig. 2D, E). Our results revealed that 27HC treatment causes increased ROS levels in LKS, HPCs, and HSCs (Fig. 2D, E). To test whether ROS level is rescued by *N*-acetyl-l-cysteine (NAC), an antioxidant that blocks ROS [52], we pre-treated BM cells with 6.2 µM 27OHChol for 24 h, followed by treatment with 1 mM NAC for 1 h (Fig. 2F and Fig. S2D). NAC treatment rescued HSPC cell number (Fig. 2F), including cKit^+^ HSPCs (Fig. S2D) in primary BM cells. This observation indicates that the number of HSPC compartments after 27HC treatment is decreased owing to the significantly increased ROS levels and apoptosis in the BM cells, partially in a ROS-dependent manner.

Interestingly, ROS can induce ER stress [53]. ER stress is a signaling pathway that occurs during ER dysfunction. ER stress induces several mechanisms including cell death [54]. A recent study has shown that 25HC induces apoptosis through the ER stress response pathway mediated by the oxysterol binding protein-related 8 (ORP8) [24]. Thus, these findings directed us to assess the ER stress response post 27HC treatment in HSPCs (Fig. 2G). Our findings revealed that 27HC treatment significantly augments the expression of C/EBP homologous protein (*Chop*) and Inositol-requiring enzyme-1*α* (*Ire1α*) in the HSPC compartments. Collectively, these data suggest that 27HC increases ROS responses and induces apoptosis through the ER stress response pathway in HSPCs.

### Impacts of 27HC in hematopoietic progenitors in vivo

**Fig. 3:**
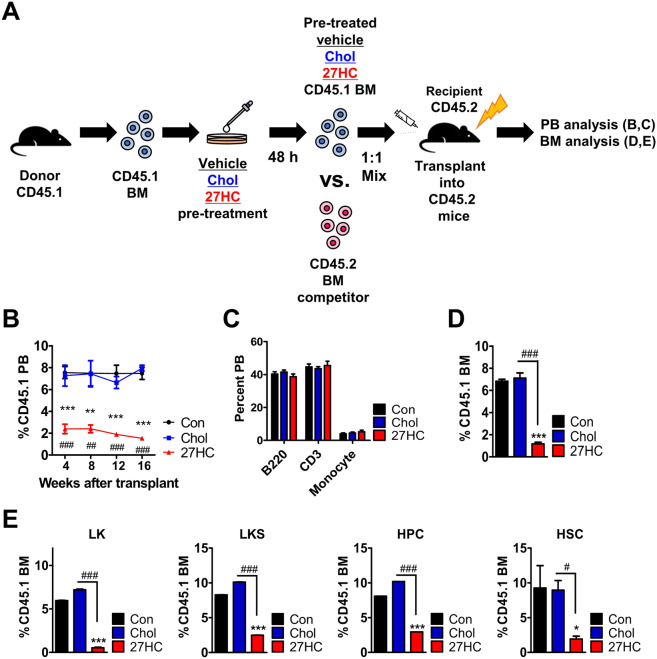
HSCs were depleted from the 27HC treated-HSPCs setting under transplantation stress. BM cells are pre-treated with 13 µM Cholesterol or 6.2 µM 27OHChol for 48 h, respectively. **A** Study overview. A 1:1 mixture of CD45.1 test cells (Control vehicle, pre-treated cholesterol, and pre-treated 27HC BM cells) were transplanted with CD45.2 competitor BM cells. **B** The peripheral blood (PB) was collected and analyzed for CD45.1 chimerism (*n* = 5). **C** The frequencies of B cells (B220), T cells (CD3), and Myeloid cells (Myelo, CD11b^+^) from the peripheral blood (PB) of transplant recipients are shown at 12 weeks after transplantation (*n* = 5). **D** The BM cells were collected and analyzed for CD45.1 chimerism at 16 weeks after transplantation (*n* = 5). **E** The percent of CD45.1 chimerism to indicated populations from the recipients were assessed from the BM cells (LK = Lin^−^Sca1^−^cKit^+^, LKS = Lin^−^Sca1^+^cKit^+^, HPC = Lin^−^Sca1^+^cKit^+^CD48^+^, HSC = Lin^−^Sca1^+^cKit^+^CD150^+^CD48^−^) (*n* = 5). Data are presented as mean ± SEM. (*p ≤ 0.05, **p ≤ 0.01, and ***p ≤ 0.001 vs. control; ^#^*p* ≤ 0.05, ^##^*p* ≤ 0.01, and ^###^*p* ≤ 0.001 vs. Chol).

27HC daily treatment did not significantly affect the numbers of HSPC in the BM under steady-state (Fig. S3 and Oguro group [37]). Also, under steady-state, 27HC treatment did not induce apoptosis in HSPCs in BM [37]. Oguro group show increased mobilization of HSCs to the spleen, only in the setting of pregnancy. To further assess the role of 27HC in HSC regeneration under stress conditions, control vehicle, pre-treated cholesterol, and pre-treated 27HC BM cells from CD45.1 mice were mixed at a 1:1 ratio with CD45.2 competitor cells and transplanted into lethally irradiated CD45.2 mice (Fig. 3A). To examine donor chimerism of test cells following transplantation, we monitored repopulation maintenance in mice for 4 months (Fig. 3B). 27HC pre-treated grafts tended to give rise to significantly lower PB chimerism (Fig. 3B) and BM chimerism (Fig. 3D) compared with control. Four months after transplantation, mice transplanted with 27HC pre-treated BM cells displayed no difference in mature blood cells compared with control in PB (Fig. 3C). In addition, mice transplanted with 27HC pre-treated BM cells displayed significantly diminished repopulation of HSPCs compared with control from the recipient mice (Fig. 3E). This was caused by a decrease in HSPC compartments of 27HC pre-treated BM cells in transplanted recipients compared with control recipients under stress conditions.

### Exogenous addition of 27HC suppresses the growth of leukemic cells

**Fig. 4:**
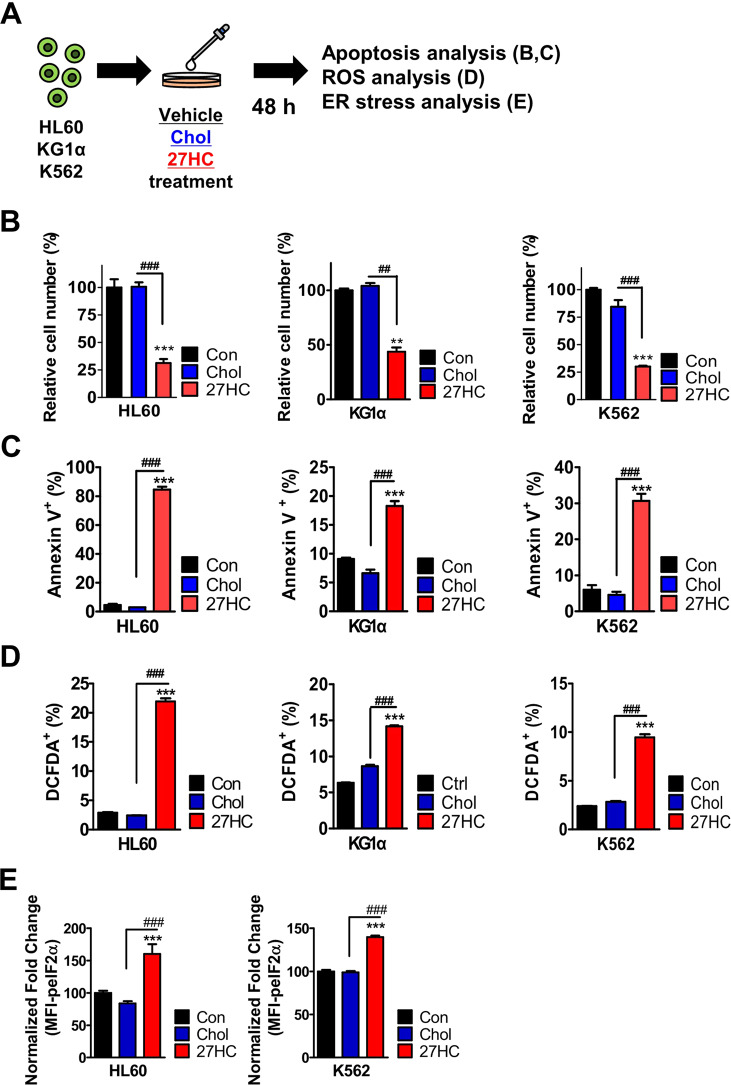
Exogenous addition of 27HC affects leukemic cell growth in vitro and in vivo. HL60, KG1a, and K562 myeloid leukemic cells are treated with 13 µM Cholesterol or 6.2 µM 27OHChol for 48 h, respectively. **A** Study overview. **B** HL60, KG1a, and K562 myeloid leukemic cells were decreased after 27HC treatment. **C** The exogenous 27HC-treated HL60, KG1a, and K562 myeloid leukemic cells showed a greater percentage of apoptotic cells than did control cells. **D** The exogenous 27HC-treated HL60, KG1a, and K562 myeloid leukemic cells showed greater ROS production than control cells. **E** 27HC increases the activity of the ER stress response in HL60 and K562 cells. Data are presented as mean ± SEM. (**p ≤ 0.01 and ***p ≤ 0.001 vs. control; ^##^*p* ≤ 0.01 and ^###^*p* ≤ 0.001 vs. Chol). (*n* = 2 independent experiments and 3 total measurements per treatment).

Several studies have demonstrated that oxysterols have pro-apoptotic and cytotoxic effects on tumor cells [55, 56]. In particular, oxysterols such as 7β-hydroxycholesterol (7βHC), 7-ketocholesterol (7KC), and 25-hydroxycholesterol (25HC) have cytotoxic effects on leukemia and lymphoma cells [57–59]. These findings encouraged us to explore the effect of 27HC on the growth of leukemic cells (Figs. 4A, B and S4A). Our results revealed that 27HC treatment arrests the leukemic cell growth in HL60, KG1α, and K562 cells (Figs. 4B and S4A). Subsequently, cell death was also determined in 27HC-treated HL60, KG1α, and K562 cells (Figs. 4C and S4B) in addition to the ROS levels (Figs. 4D and S4C). The results showed significant augmentation in the apoptosis and ROS levels in the HL60, KG1α, and K562 cells subjected to 27HC treatment. Further, we assessed the ER stress response in 27HC-treated HL60 and K562 cells (Figs. 4E and S4D). Our results revealed that the expression of peIF2α was significantly increased in HL60 and K562 cells upon 27HC treatment. 27HC induced apoptosis through the accumulation of reactive oxygen species (ROS) which activated the ER stress response. Collectively, these data suggest that 27HC increases ROS response and induces apoptosis through the ER stress response pathway.

### *CYP7B1* expression is increased (expected to have inhibition of 27HC) in acute myeloid leukemia (AML) and predictive of overall survival in AML patients

**Fig. 5:**
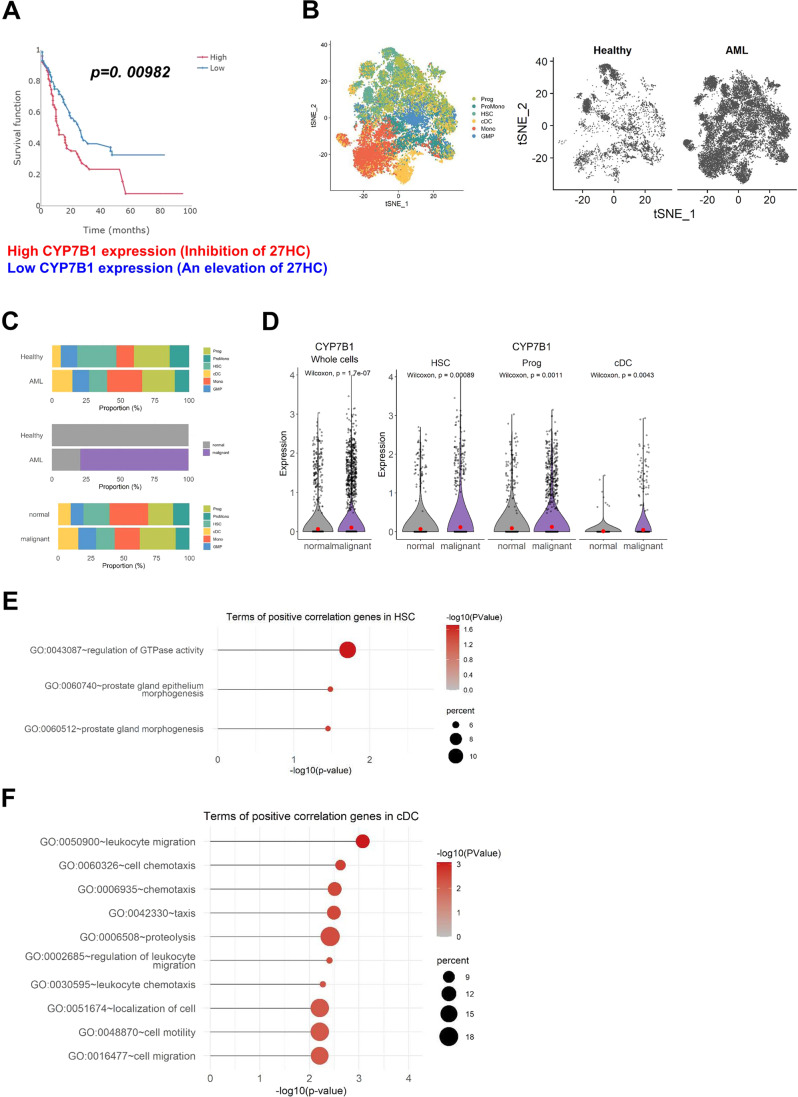
scRNA-seq analysis of acute myeloid leukemia (AML) dataset GSE116256. **A** Survival curve relative to *CYP7B1* expression (high expression (*n* = 56) and low expression (*n* = 55)) in individuals affected by acute myeloid leukemia (AML) and based on GSE12417 (log-rank test, *p* = 0.00982) (https://easysurv.net/#/app/home). **B** tSNE plot of AML patients and healthy subjects. Cells are annotated into six cell types. Granulocyte-macrophage progenitor (GMP); progenitor (Prog); promonocyte (ProMono); hematopoietic stem cell (HSC); conventional dendritic cell (cDC); monocyte (Mono). The cells from the healthy group and AML patients are located below. **C** The stacked bar plot shows the composition of cell types. Top bar plots indicate the proportion of cell types in 4430 cells from healthy donors and 17,015 cells from AML patients. In the middle, the plot exhibits components and proportions of normal cells and malignant cells in healthy donors and AML patients. At the bottom, it shows the proportion of cell types in 7956 normal cells and 13489 malignant cells. **D** Gene expression of *CYP7B1*. From left to right, plots describe expression levels of *CYP7B1* in whole-cell types, HSC, progenitor, and cDC. Expression levels are calculated as log-normalized counts and the Wilcox rank-sum test was performed to compare normal cells and malignant cells. The red dot in the plot represents the mean expression value. **E** Gene Ontology terms of *CYP7B1*-correlated genes in HSC. The top 10 biological processes of genes that are differentially correlated to CYP7B1 between malignant and normal cells are listed. The color of the dot represents the significance of the term and size refers to the percentage of enriched genes. **F** Gene Ontology analyses are similar to (**E**), but for cDC.

CYP7B1 is a 27HC metabolizing enzyme and elevations in 27HC via *Cyp7b1* deletion promote atherosclerosis in *Apoe*^−/−^ mice [31]. We first explored the overall survival of acute myeloid leukemia (AML) patients based on relative levels of *CYP7B1* expression from TCGA [42]. Individuals with high *CYP7B1* expression (expected to have inhibition of 27HC) had significantly shorter survival than those with low *Cyp7b1* expression (expected to have an elevation of 27HC) (Fig. 5A).

In addition, we explore *CYP7B1* expression in AML patients (Fig. 5B–F). To determine the expression of *CYP7B1* in AML, it might be identifiable in data generated from recent efforts to distinguish AML hierarchies [43]. Bernstein’s group showed an atlas of AML cell states by scRNA-seq. To characterize the expression of *CYP7B1* in AML, we first downloaded and explored the relevant datasets from GSE116256 [43] (Fig. 5B). The scRNA-seq data from BM cells of four healthy donors and 16 AML patients were subjected to uniform manifold approximation and projection (UMAP) analysis [60] (Fig. 5B). These populations were identified based on the expression of canonical marker genes for mature terminal lineages and genes for hematopoietic stem/progenitor cells (HSPCs). We then analyzed the composition of mature hematopoietic lineages in healthy donors and AML samples (Fig. 5C). Compared with healthy BM samples, AML has a higher proportion of conventional dendritic cells (cDCs) and monocytes and lower proportions of HSCs (Fig. 5C, top). We then distinguished the proportion of normal and malignant cells in healthy donors and AML patients, respectively (Fig. 5C, middle). We identified the proportion of cell types in normal and malignant cells from patients with AML (Fig. 5C, bottom). The malignant subset had a higher proportion of cDCs and progenitors. Further, we checked *CYP7B1* expression in AML patients. *CYP7B1* was highly expressed in the total population (Fig. 5D, left) and, in particular, *CYP7B1* displayed remarkably high expression in HSC, progenitor cells, and cDC of AML patients (Fig. 5D, right). Gene ontology (GO) analysis revealed that *CYP7B1* was associated with differentially expressed GTPase activity and leukocyte migration-related genes (Fig. 5E, F). Collectively, the observation that the expression of *CYP7B1* was significantly increased in AML patients suggests that pharmacological inhibition of CYP7B1 (expected to have an elevation of 27HC) would potentially have fewer long-term hematological side effects, particularly when used in combination with chemotherapy or radiation for the treatment of leukemia patients.

## Discussion

**Fig. 6:**
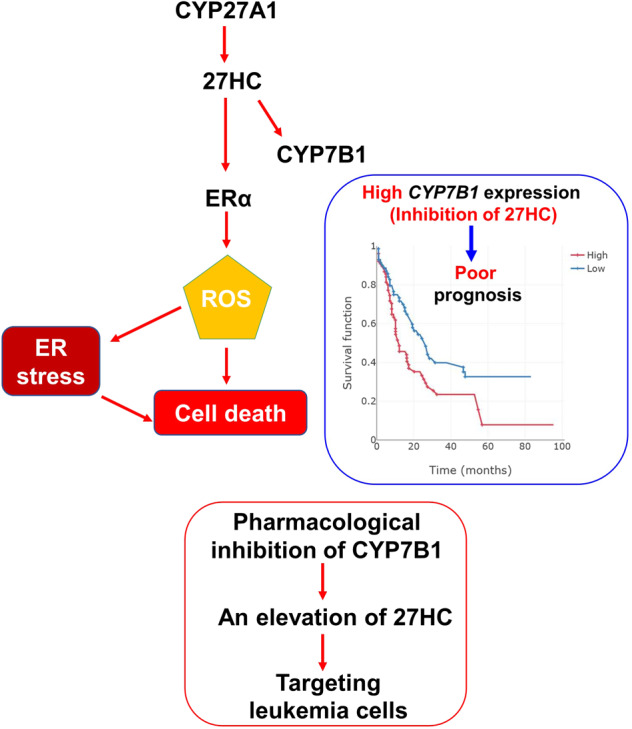
Schematic model. 27HC is the most effective oxysterol in the circulatory system and hematological malignancies. 27HC increases the ROS response and induces apoptosis through the ER stress response pathway. Pharmacological inhibition of CYP7B1 (expected to have an elevation of 27HC) would potentially have fewer long-term hematological side effects, particularly when used in combination with chemotherapy or radiation for the treatment of leukemia patients.

In this study, we have demonstrated for the first time that 27HC is the most effective oxysterol in the circulatory system and hematological malignancies (Fig. 6). We have first demonstrated that exogenous 27HC treatment results in an impaired HSPC population owing to significantly increased ROS levels, ER stress response, and apoptosis in HSPCs. We have also revealed that exogenous 27HC treatment suppresses cell growth and promotes apoptosis as well as ROS production in leukemic cells. Interestingly, we did observe decreased expression of cKit in HSPC after 27HC treatment. Recent studies reported that MED12, a component of the Mediator complex, is required for the transcriptional regulation of cKit in HSCs [61]. In addition, the CCCTC-binding factor (CTCF) is a DNA-binding zinc-finger protein and regulates the number of cKit^+^ HSC [62]. As exogenous 27HC treatment showed reductions in cKit^+^ HSPC, assessing whether 27HC directly regulates cKit transcript expression in association with the mediator and/or chromatin modifiers could prove to be of value.

ROS have high reactivity and modulate various HSC functions, including self-renewal, migration, and development [63]. A recent study has shown that a high-fat/high-cholesterol (HFHC) diet results in increased levels of ROS, and the injection of the ROS inhibitor N-acetylcysteine (NAC) attenuates HSPC expansion post-HFHC diet [64]. Elevation of ROS levels is associated with hindered HSC quiescence and self-renewal and acceleration of HSC exhaustion through the p38 MAPK pathway [4]. Several oxysterols have significant roles in the hematopoietic system and have been shown to exert cytotoxic, oxidative, inflammatory, and/or immunosuppressive effects in several cells [12, 13]. A recent study has demonstrated that 27HC induces HSC mobilization from the BM to the spleen, only in the setting of pregnancy [37]. In addition, daily treatment of 27HC does not significantly affect the number of HSPC in the BM under steady-state conditions (Fig. S3 and Oguro group [37]). Moreover, under similar conditions, 27HC treatment does not induce apoptosis of HSPCs in BM [37]. 7α-hydroxycholesterol (7αHC), 7β-hydroxycholesterol (7βHC), and 7KC are the derivatives of 7-oxygenated cholesterol. A study has shown total rescue of human retinal pigment epithelium cells upon treatment with 7βHC along with resveratrol as compared to 7βHC treatment alone [65]. 7KC promotes cell death via a caspase-dependent pathway in CML cells [59]. In this study, we have also used another oxysterol, 7αHC for comparison (Fig. S5). Our results revealed that CML cells are not as sensitive to 7αHC as they are to vehicle control and cholesterol (Fig. S5B). Furthermore, AML cells were found to be more susceptible to 27HC than 7αHC cells (Fig. S5A). However, 25-hydroxycholesterol (25HC) was found to suppress the growth of MDS cells [58]. The Tsujioka group has also reported that MDS cells respond to 27HC. A previous study showed that 7βHC and 25HC inhibit the proliferation of THP1 cells through apoptosis [66].

In summary, 27HC is indispensable for regulating pools of HSPCs and cell fate decisions and may serve as a novel therapeutic target for hematological malignancies. More immature HSPC are affected in the bone marrow by 27HC treatment under steady-state and stress conditions. However, in more mature hematopoietic populations, 27HC treatment is without significant cost to cell number or function under steady-state and stress conditions. The physiological mechanisms linking atherosclerosis, hypercholesterolemia, and hematopoiesis are the subject of ongoing research [67, 68]. For example, it may explain why patients undergoing HSC transplantation rarely experience graft failure, despite being in an extreme catabolic state [69–71]. Based on our survival and single-cell RNA-sequencing analysis, the anti-leukemic activity of 27HC points toward a novel link between ROS, ER stress, and pharmacological inhibition of CYP7B1 (expected to have an elevation of 27HC) would potentially have fewer long-term hematological side effects, particularly when used in combination with chemotherapy or radiation for the treatment of leukemia patients.

## Supplementary information

Supplemental material

Checklist

## Data Availability

The scRNA-seq dataset of bone marrow (BM) cells from AML patients and healthy controls were downloaded from the Gene Expression Omnibus (GEO) database (GSE116256).
