# Supplementary material for: Role of reactive oxygen species in regulating 27-hydroxycholesterol-induced apoptosis of hematopoietic progenitor cells and myeloid cell lines
Source: Cell Death Dis. 2022 Oct 31;13(10):916. doi: 10.1038/s41419-022-05360-0 (PMC9622808; doi:10.1038/s41419-022-05360-0)
Supplement: Supplementary file 1 — Supplemental material [file 41419_2022_5360_MOESM1_ESM.pdf]

Supplemental Information

Role of reactive oxygen species in regulating 27-hydroxycholesterol-induced apoptosis of hematopoietic progenitor cells and myeloid cell lines

#Corresponding authors
Ninib Baryawno (n.baryawno@ki.se)
Yun-Hak Kim (yunhak10510@pusan.ac.kr)
Koanhoi Kim (koanhoi@pusan.ac.kr)
Dongjun Lee (lee.dongjun@pusan.ac.kr)

Supplemental Methods and Materials

7AAD was added before FACS analysis. The following antibody cocktails/antibodies were used:

Mature Cell Lineages:

Antibody	Company	Cat
PE-Cy7-CD11b (M1/70)	Biolegend	101216
APC-Gr1 (RB6-8C5)	eBioscience	17-5931-82
PE-B220R (RA3-6B2)	BD	553089
APC-Cy7-CD3 (145-2C11)	Biolegend	100330

For HSPC analysis:

Antibody	Company	Cat
PE-Cy5-CD3 (145-2C11)	BD	555276
PE-Cy5-CD4 (RM4-5)	BD	553050
PE-Cy5-CD8 (53-6.7)	Biolegend	100710
PE-Cy5-CD19 (6D5)	Biolegend	115510
PE-Cy5-B220R (RA3-6B2)	eBioscience	15-0452-82
PE-Cy5-Gr1 (RB6-8C5)	eBioscience	15-5931-82
PE-Cy5-Ter119 (TER119)	eBioscience	15-5921-82
PE-Sca1 (D7)	eBioscience	12-5981-82
APC-cKit (2B8)	Biolegend	105812
PE-Cy7-CD150 (TC15-12F12.2)	Biolegend	115914
APC-Cy7-CD48 (HM48-1)	BD	561242
FITC-CD34 (RAM34)	eBioscience	11-0341-85
PE-Cy7-CD16/32 (93)	eBioscience	25-0161-82
APC-eFluor780-CD127 (A7R34)	eBioscience	47-1271-82

For CD45.2 Analysis:

Antibody	Company	Cat
APC-CD45.1 (A20)	eBioscience	17-0453-82
FITC-CD45.2 (104)	BD	553772

Supplemental Figure Legends

Supplementary Figure 1. Gene expression levels after administration of 27HC. (A) BM cells are treated with 13 µM Cholesterol or 6.2 µM 27OHChol for 48 h, respectively. *Erα* expression was increased in 27HC-treated $Lin^-Sca1^+cKit^+$ cells (LKS) and HPCs ($Lin^-Sca1^+cKit^+$ $CD48^+$). **(B)** *Bax* expression was increased in 27HC-treated LKS and HPCs. (**p≤ 0.01 and ***p≤ 0.001 vs. control).

Supplementary Figure 2. Exogenous addition of 27HC depletes hematopoietic stem and progenitor cells (HSPCs). (A) BM cells are treated with 13 µM Cholesterol or 6.2 µM 27OHChol for 24 h or 48 h, respectively. The frequencies of $Lin^-Sca1^-cKit^+$ cells (LK), $Lin^-Sca1^+cKit^+$ cells (LKS), HPCs ($Lin^-Sca1^+cKit^+$ $CD48^+$), and HSCs ($Lin^-Sca1^+cKit^+CD150^+CD48^-$, SLAM cells) were decreased in the BM cells after exogenous 6.2 µM 27HC treatment for 24h or 48 h. **(B)** BM cells are treated with 13 µM Cholesterol, 0.62 µM or 6.2 µM 27OHChol for 24 h, respectively. No differences in the numbers of HSPC and mature lineage cells were observed after the 0.62 µM 27HC treatment for 24h. **(C)** BM cells are treated with 13 µM Cholesterol or 6.2 µM 27OHChol for 48 h, respectively. Quantification of the $cKit^+$ cells (Lin^- gate) or $CD48^+$ cells (LKS gate) from BM HSPC after the 6.2 µM 27HC treatment for 48h, respectively. **(D)** The frequencies of $cKit^+$ HSPCs were rescued in the BM cells after 1 mM NAC treatment. (*p≤ 0.01 and ***p≤ 0.001 vs. control; $^{\#}$p≤ 0.01 and $^{\#\#\#}$p≤ 0.001 vs. Chol).

Supplementary Figure 3. Administration of 27HC *in vivo*. Wild-type mice inject 27HC by intraperitoneal administration (20 mg/kg, n=5). **(A-C)** The frequencies of $Lin^-Sca1^+cKit^+$ cells (LKS), HSCs ($Lin^-Sca1^+cKit^+CD150^+CD48^-$, SLAM cells), HPCs ($Lin^-Sca1^+cKit^+$ $CD48^+$), granulocyte-macrophage progenitor (GMP) cells ($Lin^-Sca1^-cKit^+CD34^+CD16/32^+$), common myeloid progenitor (CMP) cells ($Lin^-Sca1^-cKit^+CD34^+CD16/32^-$), common lymphoid progenitor (CLP) cells (Lin^- $Sca1^{low}cKit^{low}$ $CD127^+$), and megakaryocyte-erythroid progenitor (MEP) cells ($Lin^-Sca1^-cKit^+CD34^-CD16/32^-$) were tested in the BM cells after exogenous 27HC Intraperitoneal injection (20 mg/kg). (**p≤ 0.01 and ***p≤ 0.001 vs. control).

Supplementary Figure 4. HL60, KG1a, and K562 myeloid leukemic cells are treated with 13 µM Cholesterol or 6.2 µM 27OHChol for 48 h, respectively. (A) HL60, KG1a, and K562 myeloid leukemic cells are treated with indicated concentrations of 27HC for 48

h. **(B)** FACS plot showing the proportion of apoptotic cells in 27HC-treated HL60, KG1a, and K562 myeloid leukemic cells. **(C)** FACS plot showing ROS production in 27HC-treated HL60, KG1a, and K562 myeloid leukemic cells. **(D)** FACS plot showing the normalized fold change in MFI for peIF2α in 27HC-treated HL60 and K562 myeloid leukemic cells. (**p≤ 0.01 and ***p≤ 0.001 vs. untreated).

Supplementary Figure 5. Comparison of leukemic growth between 27HC and 7aHC in (A) HL60 cells and (B) K562 cells. HL60 and K562 myeloid leukemic cells are treated with 13 µM Cholesterol, 6.2 µM 27OHChol, or 6.2 µM 7aOHChol for 48 h, respectively. (**p≤ 0.01 and ***p≤ 0.001 vs. control).

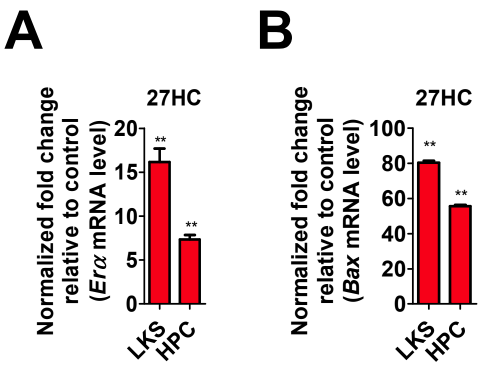
A
B
27HC
27HC
Normalized fold change relative to control (Erα mRNA level)
Normalized fold change relative to control (Bax mRNA level)
LKS
HPC
LKS
HPC

Figure S1

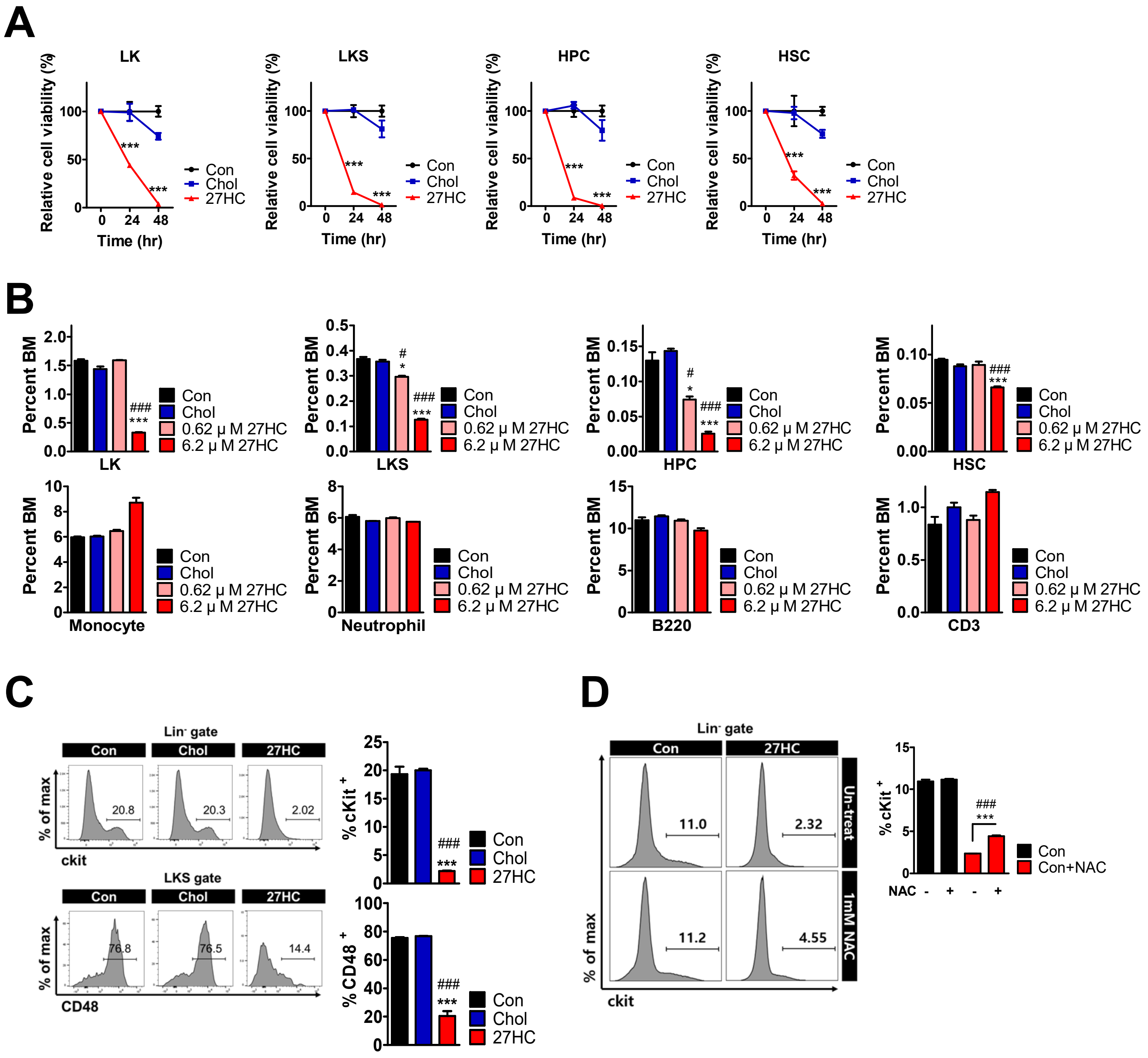
A
LK
LKS
HPC
HSC
Relative cell viability (%)
Time (hr)
Con
Chol
27HC
B
Percent BM
Con
Chol
0.62 μ M 27HC
6.2 μ M 27HC
LK
LKS
HPC
HSC
Monocyte
Neutrophil
B220
CD3
C
Lin- gate
Con
Chol
27HC
% of max
ckit
20.8
20.3
2.02
LKS gate
CD48
76.8
76.5
14.4
% cKit +
% CD48 +
D
Lin- gate
Un-treat
1mM NAC
11.0
2.32
11.2
4.55
Con+NAC
NAC

Figure S2

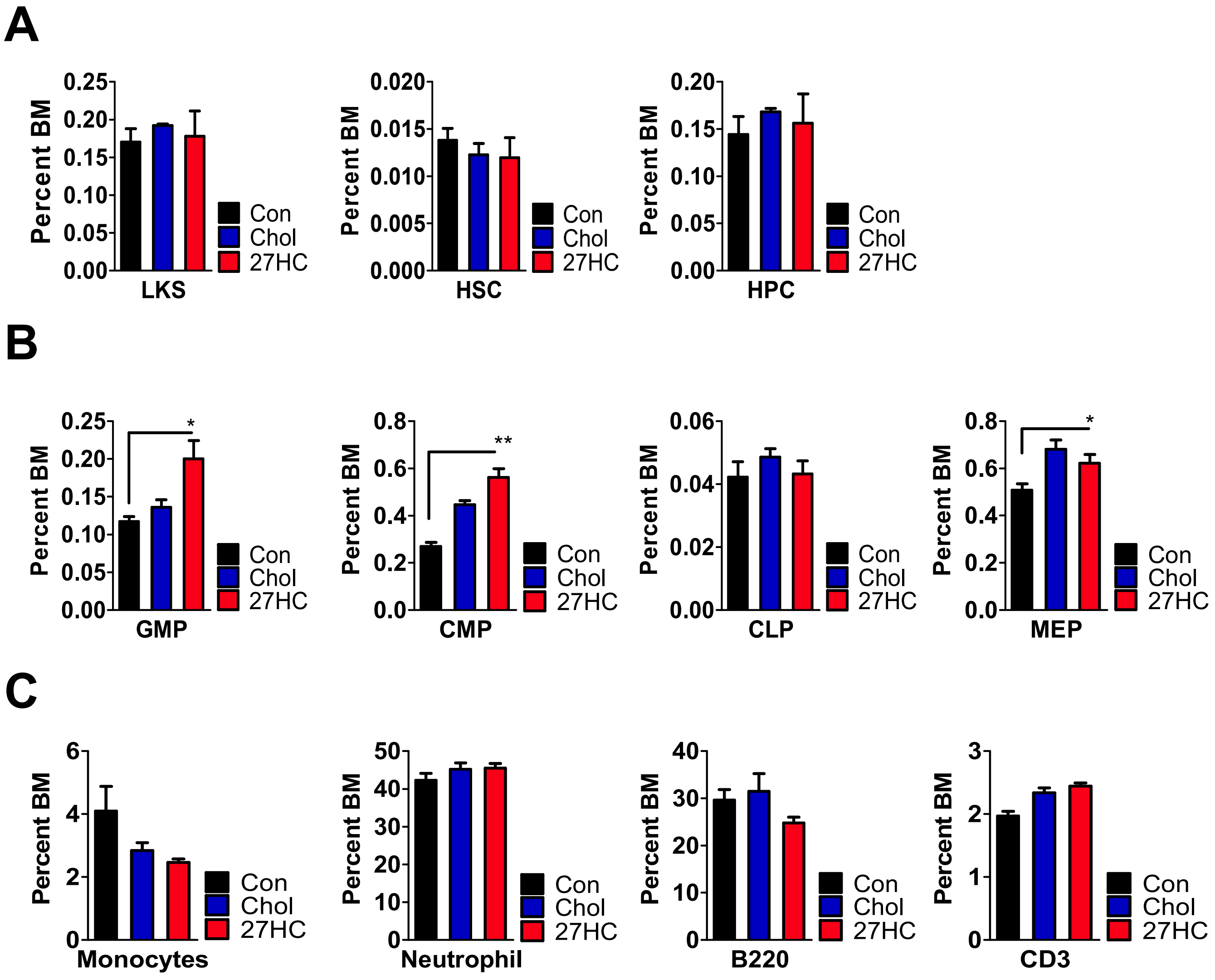
A
Percent BM
LKS
HSC
HPC
Con
Chol
27HC
B
GMP
CMP
CLP
MEP
*
**
C
Monocytes
Neutrophil
B220
CD3

Figure S3

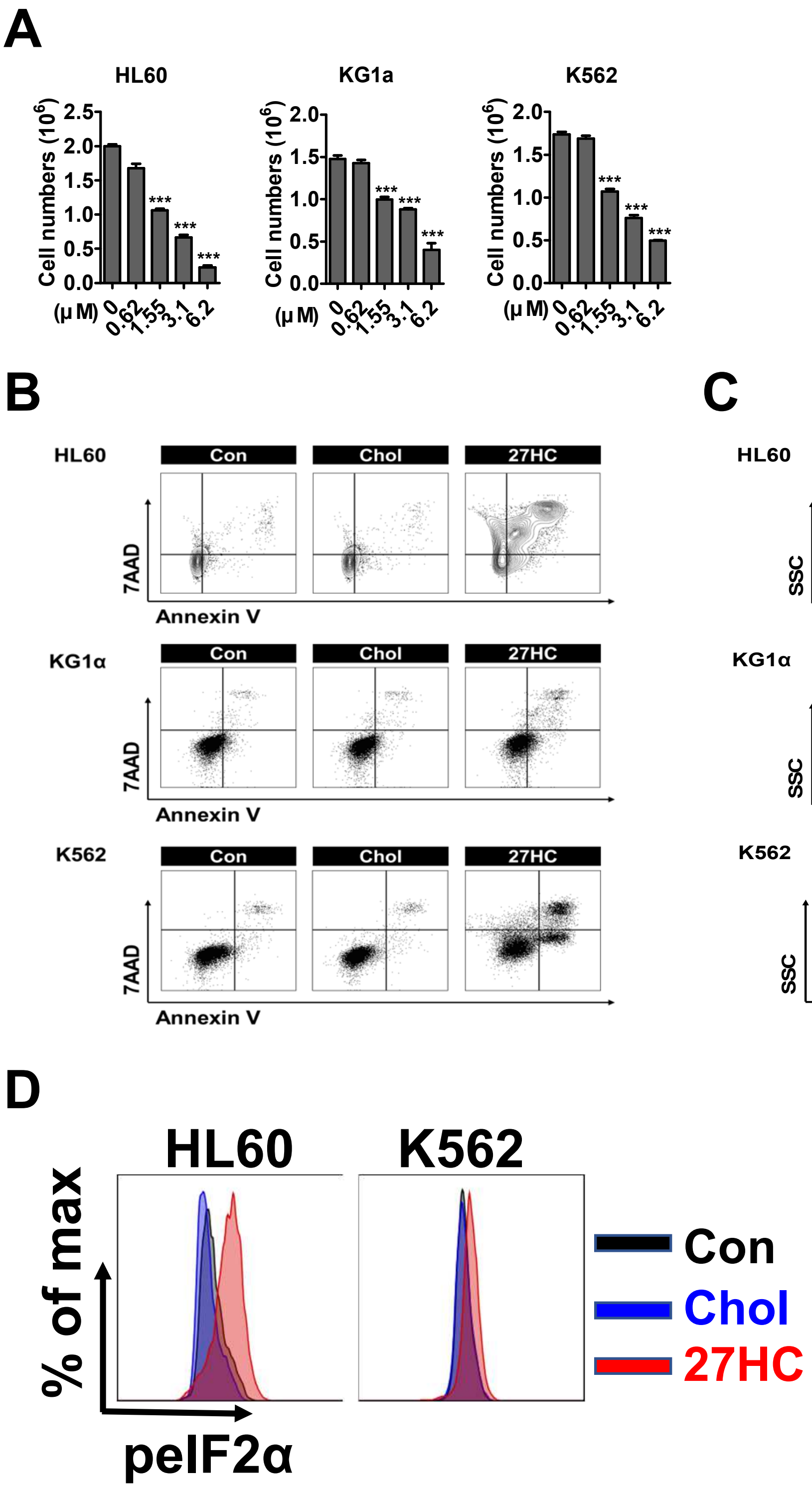
A
HL60
KG1a
K562
Cell numbers (10^6)
(μM)
0
0.62
1.55
3.1
6.2
***
B
C
HL60
KG1α
K562
Con
Chol
27HC
7AAD
Annexin V
SSC
DCFDA
2.82
2.44
22.6
6.15
8.35
14.3
2.38
2.69
10.0
D
% of max
peIF2α
Con
Chol
27HC

Figure S4

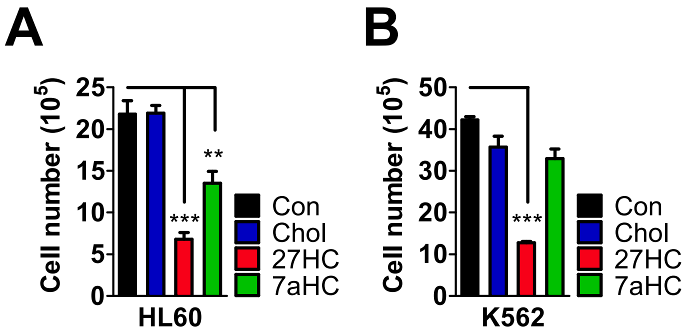
A
Cell number (10^5)
25
20
15
10
5
0
**
***
HL60
Con
Chol
27HC
7aHC
B
Cell number (10^5)
50
40
30
20
10
0
***
K562
Con
Chol
27HC
7aHC

Figure S5
